# Emerging diversity in extracellular vesicles and their roles in cancer

**DOI:** 10.3389/fonc.2023.1167717

**Published:** 2023-06-16

**Authors:** Ikjot S. Sohal, Andrea L. Kasinski

**Affiliations:** ^1^Department of Biological Sciences, Purdue University, West Lafayette, IN, United States; ^2^Purdue Institute for Cancer Research, Purdue University, West Lafayette, IN, United States

**Keywords:** extracellular vescicles, cancer, exosomes, migrasomes, exophers, metastasis

## Abstract

Extracellular vesicles have undergone a paradigm shift from being considered as ‘waste bags’ to being central mediators of cell-to-cell signaling in homeostasis and several pathologies including cancer. Their ubiquitous nature, ability to cross biological barriers, and dynamic regulation during changes in pathophysiological state of an individual not only makes them excellent biomarkers but also critical mediators of cancer progression. This review highlights the heterogeneity in extracellular vesicles by discussing emerging subtypes, such as migrasomes, mitovesicles, and exophers, as well as evolving components of extracellular vesicles such as the surface protein corona. The review provides a comprehensive overview of our current understanding of the role of extracellular vesicles during different stages of cancer including cancer initiation, metabolic reprogramming, extracellular matrix remodeling, angiogenesis, immune modulation, therapy resistance, and metastasis, and highlights gaps in our current knowledge of extracellular vesicle biology in cancer. We further provide a perspective on extracellular vesicle-based cancer therapeutics and challenges associated with bringing them to the clinic.

## Introduction

Cell-to-cell communication is central to autocrine, paracrine, and endocrine signaling, which are involved in various physiological and pathological processes. Tissue/organ homeostasis, immune function, response to stimulus, digestion, respiration, excretion, etc. are all physiological processes where cell-to-cell communication is vitally important. This exchange of information between cells is equally important in various pathological processes including cancer. While the genetic architecture of cancer cells is considered to be the driver in cancer development, cell-to-cell communication is equally essential to evade tumor suppressors, modulate extracellular matrix and immune response, resist/evade cancer therapy, and promote metastasis. Cell-to-cell communication is known to be mediated by secreted cytokines, growth factors, hormones, oligonucleotides, and other biomolecules. However, in the past decade, extracellular vesicles (EVs) have emerged as an additional important mediator" to "extracellular vesicles (EVs) have emerged as additional important mediators of cell-to-cell communication, influencing various stages of cancer progression.

EVs are a group of heterogenous membrane bound vesicles classified by their size, function, or pathway of biogenesis that contain biologically active molecules such as lipids, nucleic acids (DNAs, mRNAs, miRNAs, lncRNAs, piRNAs, etc.), and proteins (1–4). Adding to the heterogeneity, recent studies have described novel populations of EVs and extracellular nanoparticles (ENPs), such as ‘mitovesicles’ originating from mitochondria, ‘migrasomes’ formed during cell migration, ‘exophers’ involved in cellular homeostasis, ‘exomeres’, and ‘supermeres’. EVs are ubiquitously present in several biological fluids including saliva, bronchioalveolar lavage, mucus secretions, plasma, cerebrospinal fluid, amniotic fluid, breast milk, malignant ascites, and urine. As intercellular communication mediators, the secretion and content of EVs dynamically changes during homeostasis and disease progression, providing crucial molecular information regarding the health status of a certain tissue, organ, or individual. For this reason, and the ability to collect EV-containing biofluids via non-invasive or minimally invasive procedures, EVs and their contents have been proposed as prognostic, diagnostic, and predictive biomarkers in various cancers (5–18). In lung cancer, the level of EVs in the pulmonary blood strongly correlates with the clinical stage of lung cancer patients (19). For example, in individuals with stage III premetastatic NSCLC tumors elevated Tspan8 expression on serum-procured EVs is associated with lower metastasis-free survival (20). In pancreatic cancer, EV-specific GPC1 (glypican-1) and a miRNA signature (high miR-10b, miR-21, miR-30c, and miR-181a and low miR-let7a) can reliably detect early pancreatic cancer and have been shown to be superior to the standard CA 19-9 plasma test (21, 22). Several other *in vitro* and *in vivo* studies have demonstrated that EVs and their content are outstanding biomarkers for cancer progression, metastasis, and resistance to therapy, which further indicates the dynamic regulation of EV secretion and EV content during the various stages of cancer.

This review aims to provide a comprehensive overview of the role of EVs during the different stages of cancer. Firstly, the review briefly discusses emerging EV subtypes, their biogenesis and current understanding of their role in cancer. Secondly, the article discusses the role of EVs in early tumor formation, and aspects of tumor growth and survival, such as metabolic reprogramming, extracellular matrix remodeling, angiogenesis, immune modulation, and metastasis (23, 24). We discuss the underlying biology using examples from different tumor types, highlight gaps in our current knowledge and future directions, and conclude with perspectives on EV-based cancer therapies. The vast breadth of topics covered may have led to inadvertently not citing all relevant literature. We sincerely apologize to any colleagues whose related work we were unable to cite owing to space constraints.

## The emerging heterogeneity of extracellular vesicles

**Figure 1:**
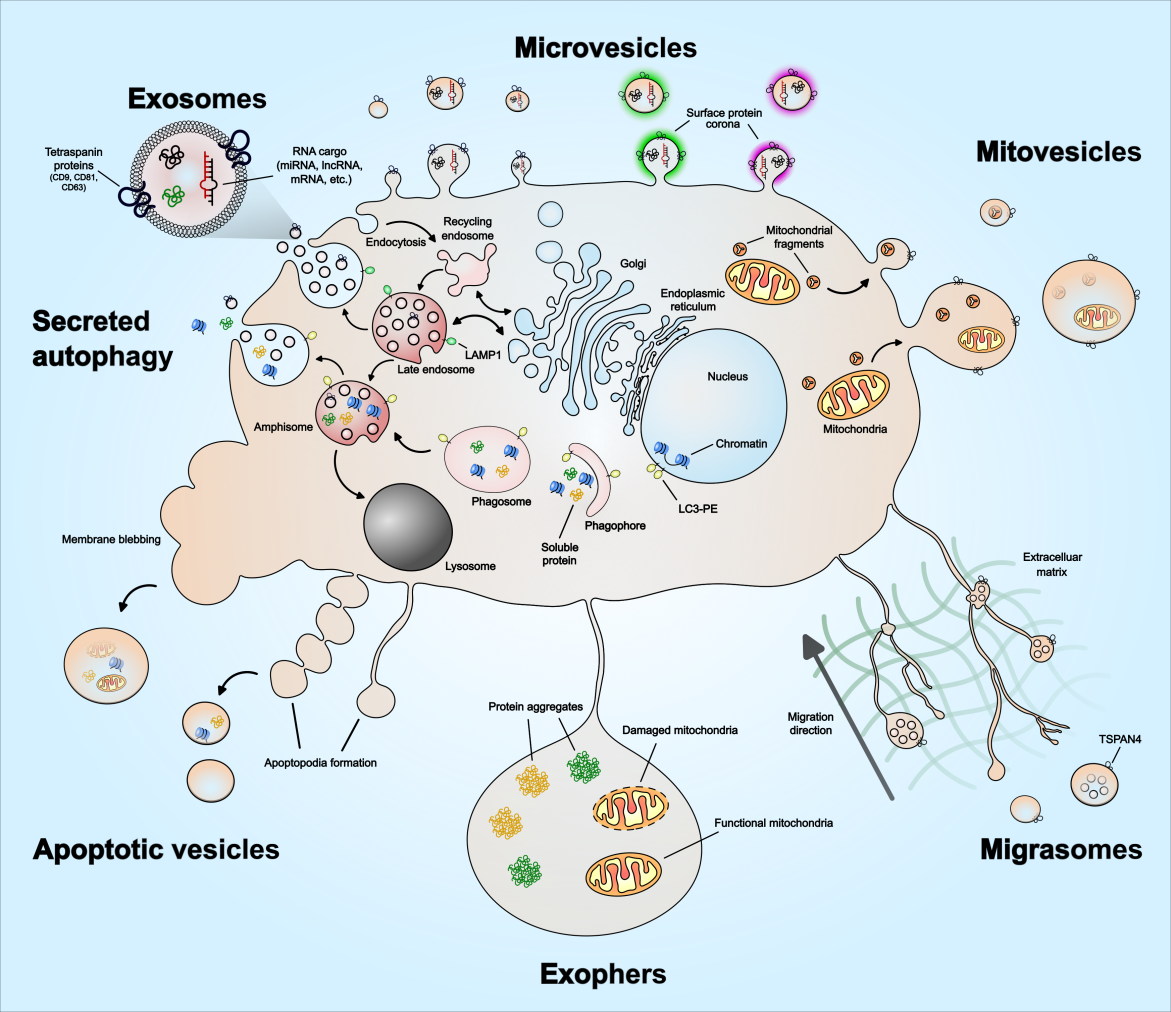
Extracellular vesicle heterogeneity and emerging subtypes. Extracellular vesicles subtypes include exosomes, microvesicles, migrasomes, mitovesicles, apoptotic vesicles, and exophers. There are other types not illustrated in the figure, which includes ‘oncosomes’ – microvesicles that contain oncogenic cargo, ‘megavesicles’ – an atypically large vesicle, synaptic vesicles and other vesicles secreted by specialized cells. Exosomes are the only EVs that form via intraluminal budding of late endosomes. The late endosomes can also fuse with a phagosome to form ‘amphisome’, which can then release soluble protein or damaged DNA cargo – a process described as ‘secreted autophagy’. Microvesicles form by plasma membrane budding and can vary in their size. Mitovesicles are EVs that can be double-layered and contain mitochondrial fragments including mitochondrial protein and lipid composition. Migrasomes form along the trailing fibers of a migrating cell and can have multi-layered vesicles or several vesicles within one. Exophers are a type of megavesicles involved in cellular protein and mitochondrial homeostasis. Apoptotic vesicles are formed during the membrane blebbing or apoptopodia formation steps of apoptosis. The size of the apoptotic vesicles depends on how they form – larger if formed via membrane blebs and smaller if formed via apoptopodia. EVs have also been described to have a protein corona on their surface that mediates angiogenesis, which is discussed in the main text. The emerging EV subtypes – mitovesicles, migrasomes, oncosomes, megavesicles and exophers, are discussed in greater detail in the main text.

EVs are recognized as a heterogenous population of vesicles that vary in their size, biological function, and origin. These include exosomes (50 nm – 150 nm), microvesicles (100 nm – 1 µm) and apoptotic vesicles (100 nm – 5 µm). In recent years, novel EV subpopulations have been identified, including mitovesicles (65 nm – 1 µm), migrasomes (50 nm – 3 µm), oncosomes (100 nm – 1 µm), megavesicles (1 µm – 10 µm) and exophers (1 µm – 50 µm). In addition, a functional surface protein corona layer has recently been described for EVs. The diversity of EV subtypes and unique features such as surface protein corona are illustrated in **Figure 1**. Furthermore, **Table 1** summarizes features of EVs unique to specific subtypes and features shared among them. As exosomes, microvesicles, and apoptotic bodies have been extensively reviewed (27, 41–44), this section discusses the emerging EV subtypes, particularly mitovesicles, migrasomes and exophers, and the current understanding of their role in cancer.

**Table 1:** Extracellular vesicle subtypes and their features.

Extracellular vesicle subtype/size	Other names	Origin	Biogenesis machinery	Cargo	Reference
Exosomes (50 nm – 150 nm)	Also referred to as ‘intraluminal vesicles’ during biogenesis	Late endosomes Multivesicular endosomes	ESCRT complexes Ceramide synthesis	Lipid bilayer, proteins, RNAs, metabolites, DNA?	(25, 26)
Microvesicles (100 nm – 1 µm)	‘Shed vesicles’, ‘Ectosomes’, ‘Synaptosomes’, ‘Microparticles’ Microvesicles carrying oncogenic cargo called ‘oncosomes’	Plasma membrane Microvilli	ESCRT complexes ARF6-GTP	Lipid bilayer, proteins, RNAs, metabolites, DNA?	(27)
Migrasomes (50 nm – 3 µm)	–	Plasma membrane Apoptopodia?	Tetraspanin-enriched microdomains Cholesterol	Double membrane, proteins, RNAs, mitochondria, DNA?	(28)
Mitovesicles (65 nm – 1 µm)	–	Mitochondria? Autophagic pathway? Apoptosis pathway?	Same as microvesicles? Apoptotic vesicles?	Double membrane, proteins, mitochondria, mitochondrial DNA, RNA	(29)
Exophers (1 µm – 50 µm)	–	Plasma membrane	?	Lipid bilayer, proteins, mitochondria, lack nucleic acids	(30)
Apoptotic vesicles (100 nm – 5 µm)	‘Apoptotic bodies’	Plasma membrane blebs Apoptopodia	Classical apoptosis machinery	Lipid bilayer, proteins, RNAs, DNAs, metabolites, organelles	(31)
Autophagic vesicles (50 nm – 150 nm)	‘Secretory autophagy-derived vesicles’	Autophagic pathway Amphisome	LC3-conjugation machinery	Lipid bilayer, proteins, RNAs, RNA-binding proteins, metabolites, organelles	(32, 33)
Necroptotic vesicles	–	Necrosome Plasma membrane Late endosomes?	ESCRT-III	Lipid bilayer, proteins, RNAs, DNAs, metabolites, organelles	(34, 35)
Ferroptotic vesicles (75 nm – 500 nm)	–	Late endosome? Mitochondria?	LPCAT3 and ACSL4 involvement	Lipid bilayer, proteins, metabolites, RNAs?, DNAs?	(36)
Pyroptotic vesicles	–	Inflammasome Plasma membrane	Caspase-1, ASC and NLRP3? ESCRT complexes?	Lipid bilayer, proteins, RNAs, DNAs, metabolites	(37)
Megavesicles (1 µm – 10 µm)	‘Large Oncosomes’	Plasma membrane?	Apoptosis?	Lipid bilayer, metabolic enzymes, cytoskeletal proteins	(38)
Exomeres^1^ (20 nm – 40 nm)	–	Autophagic pathway? Amphisome?	?	RNAs, metabolic enzymes, Argonaute proteins, lipids?	(39)
Supermeres^1^ (20 nm – 40 nm)	–	Autophagic pathway? Amphisome?	?	RNAs, metabolic enzymes, Argonaute proteins, lipids?	(40)

^1^Exomeres and supermeres are not considered vesicles as they are not reported to have a lipid bilayer. They may contain lipids and lipid complexes similar to lipoproteins.

### Migrasomes

Migrasomes were recently discovered as ~3µm diameter vesicles along the tips or intersections of trailing retraction fibers in migrating cells (28). Migrasomes are involved in cell-to-cell communication through release of enriched chemokines, morphogens, or growth factors, as carriers of damaged mitochondria, or through lateral transfer of mRNA and proteins (45, 46). A phenotype uniquely associated with migrasomes is the presence of several smaller vesicles in their lumen, similar to MVBs. While the process of migrasome formation has been observed as early as 1963, the molecular mechanism underlying migrasome biogenesis has been described only recently. A study in 2019 described the specific tetraspanin-enriched microdomains and cholesterol in migrasome membranes (47). In an overexpression screen, the study determined that 14 out of the 33 known mammalian tetraspanins could enhance migrasome formation and further demonstrated the necessity of TSPAN4 and cholesterol in migrasome biogenesis in normal rat kidney epithelial cells and human gastric carcinoma MGC-803 cells (47). However, knocking out TSPAN4 in fibroblast cell type did not impair migrasome formation suggesting involvement of other tetraspanins and proteins in migrasome biogenesis in cell types of different migration potential. Another recent study demonstrated the role of migrasomes in relaying spatiotemporal chemical information essential for organogenesis and left-right patterning during zebrafish gastrulation – a process that is characterized by extensive cell migration and differentiation (48, 49). Other physiological processes mediated by migrasomes include mitochondrial homeostasis, angiogenesis, and proliferative vitreoretinopathy, where migrasomes from retinal pigmented epithelial cells have been implicated in this rare ocular condition (50). With regard to angiogenesis, monocyte-derived migrasomes have been described to promote vasculature formation in chick embryos (51). Migrasomes have also been shown to mediate mitochondrial quality control during cell migration by accumulating damaged mitochondria inside migrasomes, a process that was found to be essential for maintaining neutrophil viability *in vivo (* 52). Despite these studies, specifics regarding migrasome biogenesis are not yet entirely clear (53). The process of migrasome budding off from the retraction fibers membrane is described to be physical i.e., as the cells migrate further away, the connections between the cell and the retraction fibers break, and the migrasomes detach from the cell. This membrane-stiffening effect that aids in the production of migrasomes is facilitated by the presence of TSPAN4 and cholesterol on the migrasome membrane (47). However, it remains unclear if there is specific membrane budding machinery involved in the detachment of migrasome from the retraction fibers, such as ESCRT as in the case of exosome and microvesicle biogenesis. While cancer cells have been described to form migrasomes (47), how the functional cargo influences tumor microenvironment as the cells migrate and invade through extracellular matrix is not yet clear.

### Mitovesicles

In 2021, D’acunzo et al. described a distinct population of EVs following a high-resolution density gradient separation of EVs isolated from murine and human Down syndrome and diploid control brains (29). The high-density fraction was enriched in double-membrane, electron-dense EVs that expressed proteins found in the mitochondrial outer membrane, mitochondrial inner membrane, and mitochondrial matrix; however, they lacked microvesicle, exosome, endocytic, and intracellular markers. The lipid composition of the EVs enriched in mitochondrial components was also similar to the mitochondrial membrane, including a high % of cardiolipin, phosphatidylcholine, and phosphatidylethanolamine, and a low % of cholesterol and sphingomyelin (29). Due to morphological features consistent with mitochondrial origin, these EVs were called ‘mitovesicles’. D’acunzo et al. found that EVs isolated from murine and human Down syndrome brains had a higher number of mitovesicles and that their protein and nucleic acid content was altered in comparison to mitovesicles isolated from diploid control brains. While migrasomes and mitovesicles share similarities such as the presence of mitochondrial components and a double-membrane (28, 29, 52), the reported size for migrasomes and mitovesicles differs, being ~3µm and 100-200nm, respectively. However, the size differences could be due to the 0.2µm-filtration step applied during the isolation of mitovesicles. In support of the similarities, other independent studies have described the presence of large (> 1µm) vesicles containing intact mitochondria (54, 55). A comparative analysis of the cargo and lipid content of migrasomes and mitovesicles would help in determining if these are indeed distinct populations. Despite the similarities in their cargo and morphological features, further studies are required to determine if migrasomes and mitovesicles share aspects of biogenesis, such as cargo trafficking, loading, and membrane budding machinery. With mitochondrial energetics and glycolysis being considered as major metabolic processes in metabolic reprogramming and cancer progression (56), discovering dysregulation of mitovesicles in cancer would not be surprising. However, as mitovesicles were recently identified, their specific role in cancer is yet to be established.

### Exophers

Exophers are a type of large EV released by plasma membrane budding that contain protein aggregates and intact organelles. Exophers were first described while observing *C. elegans* touch receptor neurons as ~4µm size vesicles outside of the cell that were found to have a biogenesis pathway distinct from exosomes (30). Exophers are proposed to be an extension of the proteostasis network to get rid of neurotoxic aggregates when proteostasis is overwhelmed by high levels of proteotoxicity. Indeed, under proteotoxic stress, the neurons that generate exophers have increased functionality in comparison to the neurons that did not. Exophers do not express apoptotic ‘eat me’-signaling phosphatidylserine indicating distinct biogenesis from apoptotic vesicles. However, it is not yet entirely clear if EV release during autophagy, a process essential for degradation/clearance of soluble and aggregated proteins, organelles, and macromolecular complexes, could also be involved in exopher biogenesis. Instead of classic removal of apoptotic vesicles, exophers are taken up by coleomocytes, mediating transfer of neuronal materials to remote cells and promoting intercellular communication. Exophers released by body wall muscles have also been shown to support embryonic growth in *C. elegans (* 57), further supported in murine models. In mice, exophers role in mitochondrial homeostasis and proteostasis have been described to support normal function of energetically high demand cardiomyocytes (58). Cardiomyocyte-derived exophers mediate removal of dysfunctional mitochondria and other material, which is phagocytosed and cleared by heart-resident macrophages. While these studies describe the functional relevance of exophers, little remains known about their biogenesis mechanisms as well as functional roles in the context of cancer. However, based on their roles in proteostasis, mitochondrial homeostasis, and supporting embryonic growth, exophers and their dysregulation are likely to play key roles in cancer cell growth and metabolism.

### Other emerging EV subtypes

Other EV subtypes that have emerged lately include EVs released during different forms of necrosis and autophagy (59, 60). In fact, for certain cell types such as monocytes, cells undergoing necrosis release more EVs than viable or apoptotic cells (59). Recent findings have also shown that cells can undergo other forms of programmed cell death such as necroptosis, ferroptosis, and pyroptosis (37). While the function of EVs released during these cell death processes are beginning to emerge, such as the contribution of ferroptosis-dependent macrophage EVs in mesothelial carcinogenesis (34, 36), the underlying molecular mechanisms of their biogenesis remain unclear.

### Extracellular nanoparticles

In addition to EVs, recent studies have identified two classes of extracellular nanoparticles (ENPs) of much smaller size (20 – 40 nm) – exomeres and supermeres (39, 40). Both types of ENPs have distinct proteomes, associated RNAs, and organ biodistribution patterns in comparison to EVs. Functionally, in colorectal cancer, cancer-derived supermeres can transfer cetuximab drug resistance from resistant colorectal cancer cells to sensitive cells. In terms of cargo, the colorectal cancer supermeres have a distinct proteome relative to EVs and have a relatively higher abundance of extracellular RNA (exRNAs), including miRNAs, than exomeres or EVs. Supermeres are highly enriched in metabolism-related proteins such as ENO2, ectodomains of clinically relevant membrane proteins such as MET, GPC1, and EGFR, and miRNA-binding proteins including AGO1, AGO2, hnRNPA2B1, and XPO5 (40). In fact, high levels of AGO1-4 in non-cellular/non-vesicular fractions have also been reported in other cancer studies (53, 61–63) and AGO2 secretion in ENPs is proposed to be a common feature of cancer cells. These studies signify the functional relevance of ENPs; however, little remains known about their biogenesis and export mechanisms. How do ENPs form? Without a delimiting membrane, how does the diverse cargo stay intact and protected? Are ENPs a type of lipoprotein synthesized by other cell types rather than via conventional hepatic lipoprotein synthesis? Are the ENP-associated miRNAs protected in a manner similar to HDL-associated miRNAs (64)? Are ENPs also present in EVs, which may explain comparable enrichment for several miRNAs in vesicular and non-vesicular fractions (65)? While many of these questions remain, recent investigations have proposed that exomeres and supermeres represent a spectrum of cellular complexes that includes intracellular U2 ribonucleoprotein, 28S rRNA and LGALS3BP ring-like decamers (66). As the review focuses on the biology of EVs in cancer, detailed discussion of ENPs is beyond the scope of this article. The reader is guided to recent discussions and reviews of ENPs by Tosar et al. and Jeppesen et al. (66, 67).

### Purification of EVs and technical challenges

The above section highlights the diversity of EV populations released by healthy and stressed cells, and their biogenesis mechanisms. It is important to note that several EV subtypes have overlapping sizes, cargo, biogenesis mechanisms, and biological functions, which leads to challenges with the isolation of pure populations of specific EV subtypes. Despite advances in EV isolation methods, the methods currently at the research community’s disposal lead to the systematic co-isolation of EVs of distinct subcellular origins. Recognizing these challenges, the International Society for Extracellular Vesicles (ISEV) issues precise guidelines on using specific EV terminology and updates them as new EV isolation and characterization technologies emerge. The most current guideline, published in 2018, states to use the term ‘EVs’ when no information is available on the isolated population (68). Additionally, the guideline urges researchers to use operational terms for EV subtypes that refer to i) physical characteristics such as size (‘small EVs’, ‘large EVs’) with ranges defined, or density (‘low-density EVS’, ‘high-density EVs’); ii) biochemical composition (‘CD81^+^ EVs’, ‘CD63-stained EVs’); or iii) description of conditions or cell of origin (‘hypoxic EVs’, ‘cancer cell-derived EVs’). The terms ‘exosomes’, ‘microvesicles’ and others should only be used when the supporting data clearly demonstrates purification of specific subpopulation or live-cell microscopy of their biogenesis.

A recent study performed a systematic literature analysis of the EV isolation methods (69). Differential ultracentrifugation continues to be the most widely used and robust method for EV isolation; however, it requires large volumes, and may lead to low EV yield or ruptured EVs. Additionally it could be difficult to compare results between studies due to different rotor types and centrifugation speed and time. An alternative to differential ultracentrifugation that enhances the purity of the crude EV pellet obtained is density-gradient ultracentrifugation, which segregates EVs by their optical densities. While the resulting EVs obtained are relatively pure, both types of ultracentrifugation procedures are highly time- and labor-intensive, limiting their use for high-throughput applications. Using size-exclusion chromatography (SEC) for EV isolation has been gaining popularity. The advantages of SEC over ultracentrifugation-based procedures are that it is easier, faster, minimal effect on EV integrity (70), there are commercial kits available (qEV columns from IZON), and it allows for scaling up using large volumes. However, SEC can often lead to lipoprotein and albumin contamination when using plasma or serum as starting material and can dilute the EV concentration requiring downstream steps to further concentrate the EVs. Conversely, using an immunocapturing method, which purifies EVs using an antibody specific to an EV surface protein, results in very high EV purity. However, this high-cost method is usually used to study specific EV subpopulations and cannot be used to study general EV population. Other EV isolation methods include precipitation-based procedures, ultrafiltration, field-flow fractionation, microfluidics-based methods, or membrane affinity methods. However, precipitation-based methods can lead to high protein aggregate contaminants and cellular toxicity (71, 72), ultrafiltration can lead to exclusion of EV populations greater than the pore size of the filter, field-flow fractionation requires specific instrumentation and cannot be used as a standalone technique (73), microfluidics-based methods requires microfluidic chip fabrication and still fails to fully separate EVs from lipoproteins (74), and membrane affinity methods can lead to lipoprotein contaminants and enrichment of larger EVs (75). To further enhance the purity of isolated EVs, recent studies have started using combined EV isolation procedures, for e.g. Ultracentrifugation + density-gradient ultracentrifugation, SEC + density-gradient ultracentrifugation, and density-gradient ultracentrifugation + ultrafiltration. Although time-consuming, combined EV isolation procedures, especially when SEC was included, were found to be superior to single EV isolation methods (69).

It is important to note that no single EV isolation method is suitable for all EV studies. An optimal EV isolation method would need to be determined based on the starting material and volume, and downstream experiments or applications. A clinical study evaluating EVs as therapeutics would require absolute high purity over yield, whereas for a study focused on EV RNAs, a good level of EV purity with some protein contamination may be acceptable. Furthermore, isolated EVs must be evaluated for EV-enriched and EV-excluded proteins and the purity must be assessed using multiple complementary techniques. It is even more important to employ these checkpoints when studying EVs in the context of cancer because the dysregulation of genes and pathways in cancer can lead to certain proteins/RNAs, that are not normally enriched in EVs, to be enriched in cancer-derived EVs. Additionally, due to inherent cellular and genetic heterogeneity of tumors, the heterogeneity in tumor-derived EV populations is even more complex. Keeping these challenges regarding EV isolation and definition of EV subtypes in mind, the next section discusses the collective role of EVs in cancer with only a few examples discussing specific subtypes when comprehensive characterization data is available.

## Extracellular vesicles in cancer – from initiation to metastasis

**Figure 2:**
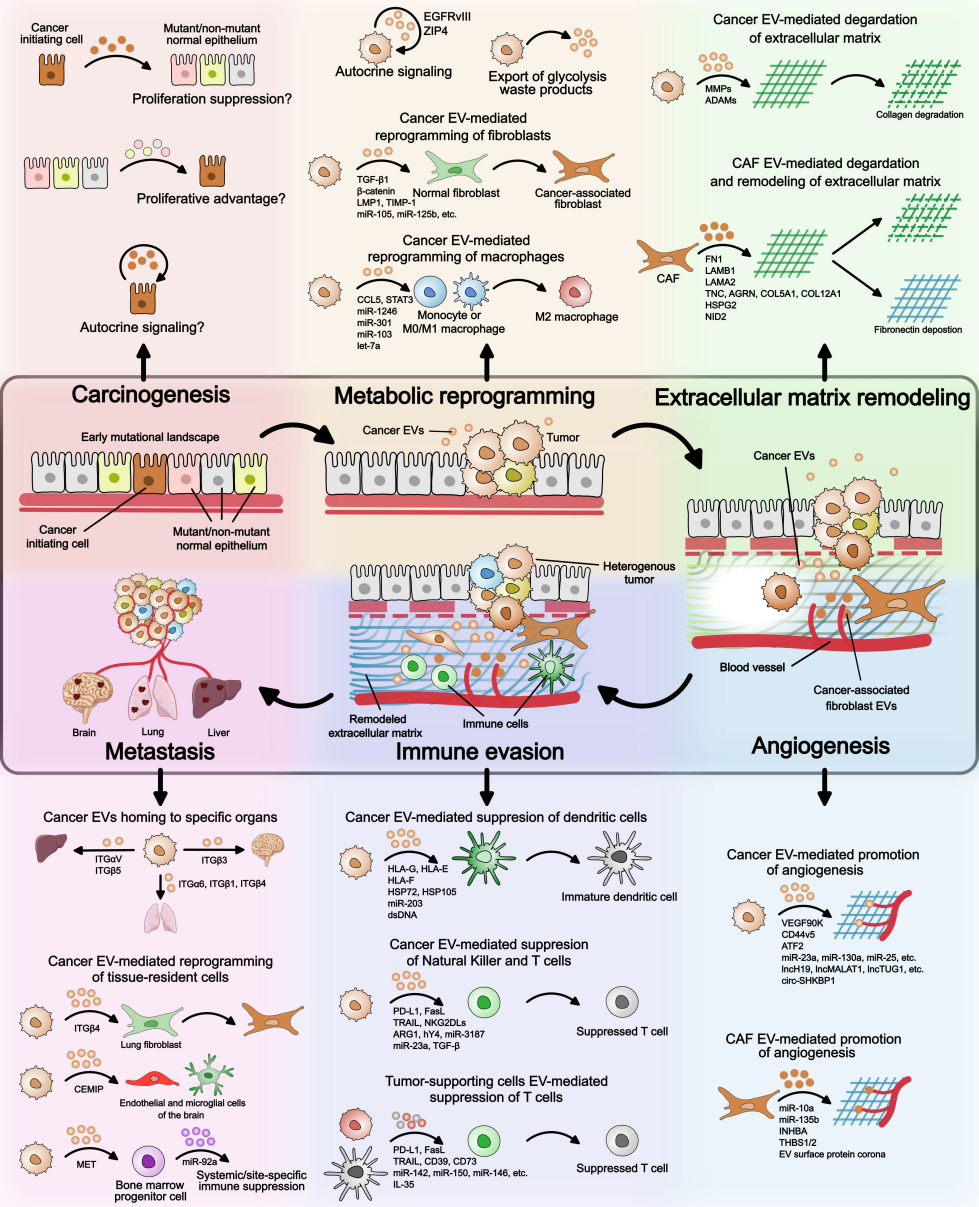
Extracellular vesicles’ role in cancer. An overview of the EV mode of action at different stages of cancer – (i) carcinogenesis, (ii) metabolic reprogramming, (iii) extracellular matrix remodeling, (iv) angiogenesis, (v) immune evasion, and (vi) metastasis. During carcinogenesis, it is not known if EVs provide proliferative advantage to cancer initiating cell over neighboring mutant/non-mutant normal epithelium. Possible mechanisms by which EVs can confer proliferation advantage to precancerous cells are illustrated. Regarding metabolic reprogramming, cancer EVs have been shown to reprogram normal fibroblasts and monocytes/macrophages to tumor-supporting phenotypes. Whether EVs from cancer initiating cell or other neighboring cell types facilitate reprogramming of cancer cells remains to be seen. Extracellular matrix remodeling and angiogenesis can be mediated by both cancer cell EVs and cancer-associated fibroblast EVs. The EV cargo involved is highlighted in the figure and detailed in **Table 2**. In immune evasion, cancer EVs suppress the maturation of dendritic cells, promote macrophage differentiation to M2 state, and directly inhibit T cell function. T cell function is also suppressed by immature dendritic cell- and M2 macrophage-derived EVs. During cancer metastasis, integrin expression on the surface of cancer EVs determines homing to specific organs. Cancer EV cargo can also reprogram tissue-resident cell types such as lung-resident fibroblasts and brain-resident endothelial and microglial cells. Furthermore, cancer EVs can suppress differentiation of bone marrow progenitor cells leading to systemic of site-specific immune suppression.

The transition of a singular cancer cell to a multicellular tumor, and later to a multi-tumor metastatic disease is a gradual, biologically demanding process. It not only requires proliferative advantage over the normal counterparts at the single cell stage but also requires survival mechanisms, as the host microenvironment and immune system resist the growing tumor. While epigenetic changes and mutations in the genetic code are known to confer proliferative and survival features, the process also requires constant communication with the surrounding cellular and acellular microenvironment. This constant sending, receiving, and sensing of information is mediated via EVs and secreted factors. The diverse roles of EVs during various stages of cancer progression are illustrated in **Figure 2** and summarized in **Table 2**. Here, we will discuss, with examples, the recent advances in how EV-mediated cell-to-cell communication facilitates cancer initiation, tumor growth, survival, and metastasis, including major gaps in our current understanding of EV biology in cancer.

**Table 2:** Diverse roles of extracellular vesicles during different stages of cancer progression.

Pathological feature	Mode of EV action	Cargo involved	Reference
Cancer initiation	Cancer EVs inhibit proliferation of adjacent normal epithelium?	–	
	Cancer EVs promote immune evasion in high surveillance sites?	–	
	Cancer EVs promote cancer proliferation via autocrine signaling?	EGFRvIII? ZIP4?	(76, 77)
Precancerous inflammation	EV-mediated crosstalk between microbiome and immune cells?	PAMPs? DAMPs?	(78, 79)
	EV-mediated crosstalk between immune, stromal, and other cells	IL-10?	(78, 80)
Metabolic reprogramming	Increased proliferation via cancer EV-mediated autocrine signaling	EGFRvIII, ZIP4	(76, 77)
	Cancer EV-mediated export to prevent accumulation of waste products produced during aerobic glycolysis	GLUT1, PKM2, lactic acid, proteins related to glycolysis I, gluconeogenesis I and the pentose phosphate pathway	(81–83)
	Secreted autophagy	–	(32, 33)
	Cancer EV-mediated reprogramming of normal fibroblasts to cancer-associated fibroblasts	Signaling factors/proteins – TGF-β1, β-catenin, IL-6, p-STAT3, Wnt2B, LMP1, TIMP-1, COL6A1, Lin28b, ITGβ_4_ miRNAs – miR-105, miR-125b, miR-130b-3p, miR-146a-5p, miR-1249-5p, miR-6737-5p, miR-6819-5p, miR-155-5p, miR-27a, miR-192, miR-215, miR-142-3p, miR-155, miR-210, miR-375, and miR-21 lncRNA – lncGm26809	Reviewed in (84)
	Cancer EV-mediated reprogramming of monocytes/macrophages to M2-macrophages	CCL5, STAT3, miR-1246, miR-301a-3p	(85–88)
Extracellular matrix remodeling	Cancer EV-encapsulated release of proteolytic enzymes	MMP14, MMP9, MMP13, MMP1, and MMP3, ADAM10, ADAM15, ADAM17 and ADAMTS5	(89–96) and reviewed in (97)
	EVs secreted by reprogrammed cancer-associated fibroblasts	FN1, LAMA2, BGN, EFEMP2, TNC, LAMB1-1, HSPG2, COL12A1, AGRN, NID2, COL5A1	(94, 98)
Angiogenesis	Cancer EVs uptake by endothelial cells	Proteins – VEGF_90K_, ASPH, ICAM1, CD44v5, ROCK1/2, ATF2, soluble E-cadherin miRNAs – miR-23a, miR-130a, miR-25-3p, miR-26a, miR-182-5p, miR-21, miR-210, miR-9 lncRNAs – lncH19, lncMALAT1, lncTUG1, lncp21, lncGAS5, lncAHIF, lncHOTAIR, lncCCAT2, lncPOU3F3 circular RNAs – circ-SHKBP1 and circRNA-100338 Secreted autophagy cargo - Cathepsin B	(60), reviewed in (99)
	EVs secreted by reprogrammed cancer-associated fibroblasts	miR-10a-5p, miR-135b-5p, INHBA, and THBS1/2 Surface protein corona?	(100–103)
Immune modulation	Immunosuppression by cancer EVs	Dendritic cell suppression – HLA-G, HLA-E and HLA-F, HSP72, HSP105, miR-203, and dsDNA T cell suppression – PD-L1, FasL, TRAIL, NKG2DLs, ARG1, hY4, miR-3187-3p M2 macrophage polarization – miR-103a, let-7a NK cell suppression – miR-23a, TGF-β	(104–113) (114, 115)
	Immunosuppression *via* EVs released by tumor-supporting cells	PD-L1, FasL, TRAIL, CD39, CD73, miR-142-3p, miR-150-5p, miR-146a-5p, let-7d, miR-9, miR-330, miR-503, and inducible nitric oxide synthase mRNA, IL-35	(116–119) (120)
	Immune activation by cancer EVs	Tumor-associated antigens (TAAs), DAMPs, MUC1, NOX2, ROS, mtDNA, BAG6	Reviewed in (121)
	Immune activation by EVs released by mature dendritic cells, natural killer cells and macrophages	Dendritic cell EVs – IFN-γ, HLA-DQ, melanoma-associated antigen 3 (MAGE-A3), MART1, glycoprotein 100 (gp100), or HPV16 E7, alpha-fetoprotein (AFP), ovalbumin, lipopolysaccharides Natural killer cell EVs – FasL, TNF, TRAIL, NKp46, NKp30, Macrophage EVs - hyaluronic acid (HA), 3-(diethylamino)propylamine, monophosphoryl lipid A, and MUC1 T cell EVs – IFN- γ, mtDNA, tRNA fragments, PD-1	(116, 122–124), reviewed in (121)
	Immune modulation by surface protein corona	Cytokines attached to the surface of T cell EVs?	(103, 125)
Therapy resistance	EV-mediated drug export/efflux	Mitoxantrone, cisplatin Mediated by intracellular ABCG2, ABCA3, and P-glycoprotein.	(126, 127)
	Cargo transfer from resistant to sensitive cells	Proteins – MRP1, UCH-L1, PDGFRβ, PTPRZ1-MET fusion protein, ALDOA, ALDH3A1, PKM2 miRNAs - miR-222, miR-96, miR-100-5p, miR-146a-5p, miR-155, miR-145, miR-34a lncRNAs - lncUCA1, lncARSR	(114, 115, 128–130)
	Cargo transfer from tumor-supporting cells	Protein – Annexin A6 miRNAs – miR-21, miR-92a-3p lncRNAs – lncH19, lncCCAL	(131–136)
	Sequestration of the drug via EV surface cargo	HER2, CD20, PD-L1?	(120, 137)
	EVs contribution to physical barriers	Fibronectin, Lactic acid?	
Metastasis	Cancer EVs homing to specific organs	Lung - ITGα_6_, ITGβ_1_, ITGβ_4_ Liver – ITGβ_5_, ITGα_v_ Brain – ITGβ_3_	(23)
	Cancer EVs reprogram normal fibroblasts in the lung	ITGβ_4_	(23)
	Cancer EVs reprogram endothelial and microglial cells in the brain	CEMIP	(138)
	Cancer EVs reprogram bone marrow-derived cells to promote systemic or site-specific immunosuppression	MET	(139)
	EVs secreted by reprogrammed bone marrow-derived cells	miR-92a	(140)

### Cancer initiation

#### Early mutational landscape and competitive advantage

During the past two decades, it has become apparent that mutations in normal cells are necessary, but not sufficient, to promote tumorigenesis. Somatic mutations in cancer-driver genes are quite frequent, and often more prevalent, in phenotypically normal adult human epithelia of esophagus, skin, endometrium, lung, bladder, and colon than in neoplastic lesions (141–149). In the early stages of solid tumor tumorigenesis, several microscopic lesions are formed but most of these lesions are eliminated through competition with mutant clones in the adjacent normal epithelium (150). The survival and proliferation of early micro-tumors depends not only on the mutations that they carry, but also on additional transformations that confer a competitive advantage over the adjacent normal tissue, such as increased proliferation or the ability to secrete factors, including EVs, that suppress the proliferation of adjacent normal tissue. The lesions that persist continue to grow in size, develop an intricate vascular network, and eventually become squamous cell carcinoma (150, 151). Additionally, to maintain homeostasis, the immune system continually detects and destroys abnormal/malignant cells through ligands expressed on their surface, which the early tumors must evade/suppress. While lumen shedding of early lesions in esophageal epithelium do not show extensive involvement of antitumor immunity, perhaps due to low immune surveillance of the tissue site, lesions in epithelial sites that are in close contact with immune cells such as alveolar epithelium and Peyer’s patches in gut epithelium, as well as blood malignancies that usually originate in the bone marrow, must evade immune challenge during the initiation phase.

Clear evidence supports the contribution of EVs and their cargo during the early stages of cancer, firstly as evidenced by their extensive utilization as biomarkers of early-stage cancer detection. For instance, in a clinical study that evaluated the presence of mutant KRAS in plasma-derived EV DNA of early-stage pancreatic ductal adenocarcinoma (PDAC) patients, 66% of the patients (22/33 patients) were positive for mutant KRAS whereas only 7.4% of age-matched control samples were positive (152). More importantly, following resection of the tumor in early-stage patients, the detection rate for mutant KRAS in plasma-derived EV DNA dropped to only 5% (1/20 patients). Other studies have also demonstrated the utility of plasma EVs for pancreatic cancer diagnosis (12, 153). While the findings indicate that early-stage PDAC tumors alter the profile and content of plasma EVs, it remains unclear if it is because of direct release of PDAC-derived EVs in the blood or indirect release from another cell type following PDAC EV-mediated signaling or secreted factor-mediated signaling. While the above examples are based on early-stage tumors, the role of EVs in several aspects of the tumor initiation process remain largely underexplored. Do EVs secreted by early micro-tumors lead to a competitive advantage over mutant clones in the adjacent normal epithelium? In high immune surveillance sites of epithelium, do EVs released by early lesions facilitate suppression/evasion of immune recognition? Similarly, in hematologic malignancies, do EVs released by cancer initiating cells affect other immune cells, enabling immune suppression or evasion? Does secretion, including EVs, from early micro-tumors in epithelial tissue or cancer initiating cells in blood malignancies influence EV release from adjacent normal cells, further supporting tumor initiation and establishment? Answers to these questions will provide key information that will help to understand the nature and dynamics of EV-mediated cell-to-cell communication in tumor initiation and will present novel strategies to therapeutically target the tumorigenic process.

#### Precancerous inflammation

As immune surveillance plays a critical role in elimination of abnormal cells and early micro-tumors during the initiation phase, physiological conditions that suppress the immune system such as obesity and inflammation can act as precursors to cancer initiation. Some of the notable examples include inflammatory bowel disease (IBD), chronic hepatitis, Helicobacter-induced gastritis, or shistostoma-induced bladder inflammation, which increase the risk of colorectal cancer (CRC), liver, stomach, or bladder cancer, respectively (154). IBD occurs as a result of dysregulation of gut homeostasis, which relies on the crosstalk between microbiota, the epithelial barrier, and the local immune system to maintain tolerance towards normal microbiota and food proteins but also to initiate efficient immune responses towards potential pathogens (155). Immuno-tolerance in the gut is mediated by several mechanisms, one of which is the immunosuppressive activity of EVs secreted from epithelial cells. Indeed, transfer of EVs from healthy intestine into mice with IBD reduced inflammation and severity of the disease (156) whereas, reverse transfer of EVs from IBD patients to human colonocytes (DLD-1) induced a pro-inflammatory response (157), highlighting the functional role of EVs in intestinal inflammation. While the specific contribution of EVs in precancerous inflammation is yet to be established, the study from Yang et al. is a notable example of how crosstalk between gut microbiota, colon epithelial cells (CECs), and macrophages facilitates precancerous inflammation in the colon, which then leads to tumorigenesis (78). Macrophages are known to exhibit tumor-promoting activity in advanced cancers (discussed below), but this study showed how inducing colon inflammation enriches for LPS-producing gram-negative microbiota, which in turn activates TLR4 in CECs and increases the expression of CCL2, a chemokine. CCL2 expression mediates recruitment of a subtype of macrophages, called monocyte-like macrophages, through CCL2/CCR2 ligand-receptor interaction. The recruited monocyte-like macrophages facilitate a chronic precancerous inflammatory microenvironment, which drives epithelial cell proliferation, tumorigenesis, and neoplasia. Recently, extensive remodeling of the extracellular matrix (ECM) was also shown to mediate inflammation-driven colon cancer (80). In this work, activation of the transcriptional regulator, heat shock factor 1 (HSF1) in colon fibroblasts was reported to play a crucial role in ECM remodeling, whereas loss of stromal HSF1 prevented ECM remodeling and progression to colon cancer. The studies discussed above emphasize the significance of intercellular crosstalk and crosstalk between microbiota and other cells. Future studies that evaluate functional EV transfer across cell-to-cell crosstalk models, such as epithelial/macrophage, epithelial/fibroblast and epithelial/immune cell, or others, and microbiota/cell crosstalk will help establish the specific roles of EVs in precancerous inflammation.

### Tumor growth and survival

As proposed by Hanahan and Weinberg, tumors are more than just insular masses of proliferating cancer cells (158). Instead, they are complex tissues composed of multiple distinct cell types that participate in heterotypic interactions with one another. During the ensuing two decades, the various distinct cell types have been extensively studied for their contribution to the tumor microenvironment, in turn promoting the hallmarks of cancer. Cancer cell-derived EVs act as mediators of the crosstalk between cancer cells and the distinct cell types to maintain, as well as evolve, the tumor microenvironment pursuant to the needs of a growing tumor. Using physiologically-relevant examples from recently published literature, we will discuss how cancer cell-derived EVs mediate several aspects of the tumor microenvironment such as extracellular matrix remodeling, angiogenesis, metabolic reprogramming, and immunosuppression.

#### Metabolic reprogramming

Oncogenic mutations during the early tumorigenesis process alter the metabolic properties of cancer cells. It is well known that in tumors, and other proliferating or developing cells, there is a dramatic increase in the rate of glucose uptake and lactate production via glycolysis. The preference to perform glycolysis instead of oxidative phosphorylation in the presence of oxygen is termed the ‘Warburg effect’ or ‘aerobic glycolysis’. While several different explanations have been proposed for the function of Warburg effect (159), the phenomenon results in high amounts of glucose uptake, increased lactate production, and acidification of the tumor microenvironment. This altered glucose metabolism has been shown to modulate ROS production and mediate changes in the chromatin state (160, 161). Additionally, the acidic niche formed from this process has been linked to ECM modulation by activation of matrix metalloproteases, controlled growth factor release, M2 polarization of macrophages, and reduced infiltration and effector functions of T cells (162), which are discussed later.

EVs resulting from metabolic reprogramming of cancer cells can modulate several tumorigenic processes such as ECM remodeling, initiating intercellular crosstalk to promote tumor microenvironment, inducing immunosuppression, and others, which are discussed in later sections. A few studies have shown that EVs from cancer cells can also contain cargo related to aerobic glycolysis, such as the glucose transporter GLUT1, pyruvate kinase PKM2, and other proteins related to glycolysis I, gluconeogenesis I, and the pentose phosphate pathway (81–83). Upregulation of the GLUT1/PKM2 metabolic loop in T-cells can promote nuclear translocation of PKM2, where it acts as a transcriptional regulator to promote release of proinflammatory cytokines, contributing to the immunosuppressive tumor microenvironment. Cancer EVs can also signal in an autocrine manner by delivering constitutively active mutant EGFR (EGFRvIII) or the zinc transporter, ZIP4 to promote their own proliferation (76, 77). However, these studies are correlative, and it is not yet known if increased aerobic glycolysis in cancer cells affects EV release and their cargo. Moreover, these studies report the aftereffects of metabolic reprogramming. Whether EVs are involved in the steps preceding early metabolic changes remains unclear. Mutations in oncogenes are often considered critical for metabolic reprogramming of cancer cells, however, similar mutations also appear in phenotypically normal epithelia (141–149). Determining how EVs ‘drive’ or ‘facilitate’ metabolic reprogramming also presents a therapeutic avenue to inhibit tumor progression at the early stage. Another question that remains unexplored is how the acidic microenvironment influences the stability and biological function of EVs. To prevent accumulation of waste products during aerobic glycolysis, cancer cells actively undergo autophagy and secrete EVs (32). In bladder cancer, autophagic EVs released by cancer cells have been described to promote angiogenesis through EV-mediated delivery of cathepsin B in endothelial cells (60). Increased cathepsin B in endothelial cells caused activation of TPX2-mediated phosphorylation of the AURKA-PI3K-AKT axis, which upregulated VEGFA expression (60). However, the link between metabolic reprogramming and increase in autophagic EV secretion is not entirely clear and requires further investigation. A recent study on metabolic turnover rate of primary and metastatic tumors determined that despite increased glycolysis in primary tumors, ATP production is slower than the adjacent normal tissue (163). On the contrary, metastatic tumors had higher oxidative phosphorylation rates and ATP levels. Whether the EV secretion rate is influenced by endogenous ATP levels remains to be seen. We previously reported that not all lung cancer cell lines have higher EV secretion rate than their normal counterparts – a common misconception in the field of EV biology. However, cell lines which did have higher EV secretion rate were the only ones capable of promoting cancerous phenotypes in non-tumorigenic epithelial cells (164). Evaluating endogenous ATP levels in low and high EV secretion rate cells can help determine if ATP levels modulate EV secretion.

#### Extracellular matrix remodeling

The extracellular matrix (ECM) is a three-dimensional, acellular structure that is present in all tissues. In mammals, the ECM is composed of ~300 proteins, which includes proteins such as collagen, fibronectin, proteoglycans, and glycoproteins (165). The ECM is mainly of two types – the stromal matrix, which surrounds cells and tissues to provide structural scaffolding, and a basement membrane, which is a specialized form of ECM that separates the epithelium and endothelium from the surrounding stroma. The ECM components constantly interact with epithelial cells by serving as ligands for cell receptors such as integrins that regulate various cell functions including adhesion, migration, proliferation, apoptosis, survival, or differentiation. The ECM can also contribute to tissue homeostasis by sequestering signaling molecules such as growth factors, chemokines, cytokines, and others. Cells are constantly rebuilding the ECM through synthesis, degradation, remodeling, and chemical modification (166). In early tumorigenesis when the cancer cells are in the epithelial lumen, cancer EVs can modulate epithelial barrier properties to provide access to the underlying stromal ECM (164). One of the primary cell types involved in stromal ECM remodeling are fibroblasts, which are reprogrammed in cancer to support tumor progression. During tumor growth, the stromal ECM undergoes drastic remodeling from a glycine-, proline- and hydroxyproline-rich collagen type I ECM to a fibrillar matrix composed of fibronectin and tenascin. Invasive carcinomas have been shown to disrupt the continuity of the basement membrane, which is primarily composed of non-fibrillar collagen type IV and laminin, promoting intravasation and metastatic spread (167).

EVs have been shown to remodel stromal ECM through two broad mechanisms – (i) secretion of proteolytic enzymes, such as matrix metalloproteinases (MMPs) that degrade collagen I-rich ECM; and (ii) recruitment and reprogramming of normal fibroblasts to cancer-associated fibroblasts (CAFs) to remodel ECM through CAF-derived EVs. Firstly, proteolytic enzymes, soluble or membrane-bound, that are secreted in or on cancer-derived EVs include MMPs – MMP14, MMP9, MMP13, MMP1, and MMP3 (89–92, 168), and the ADAM family of disintegrins and metalloproteases – ADAM10, ADAM15, ADAM17 and ADAMTS5 (93–96). The function and role of these proteolytic enzymes in cancer is summarized by Nawaz et al (97). Releasing membrane-bound MMPs, such as MMP13, in nano-sized vesicles presents an efficient way to remodel ECM during early stages when the ECM is highly dense and likely inaccessible to invading cells. Indeed, EVs have been observed to readily diffuse through an otherwise spatially confined nanoporous matrix (169). The degradation of collagen I-rich ECM leads to recruitment of fibroblasts, which in a normal repair process deposit a fibronectin-rich ECM that is eventually replaced by collagen to restore ECM integrity. However, the constant ECM degradation by cancer EVs likely results in perpetual fibronectin deposition. This constant involvement of fibroblasts in ECM rebuilding initiates EV-mediated cross-talk between cancer cells and fibroblasts which results in reprogramming of normal fibroblasts to CAFs via signaling factors (TGF-β1, β-catenin, IL-6, p-STAT3, Wnt2B, LMP1), miRNAs (miR-125b, miR-130b-3p, miR-146a-5p, miR-1249-5p, miR-6737-5p, miR-6819-5p, miR-155-5p, miR-27a, miR-192, miR-215, miR-142-3p, miR-155, miR-210, miR-375, and miR-21) and others (TIMP-1, COL6A1, Lin28b, lncRNA Gm26809) (84). The increased activity of CAFs results in extensive changes in the tumor’s mechanobiological properties that contributes to increased stiffness of tumors, which feeds back to increase tumor invasiveness and reduce therapy efficacy (170). These biomechanical signals from the tumor’s physical environment further modulate signaling pathways in cancer cells and CAFs, termed mechanotransduction. Mechanotransduction is well known to cause activation of transcription factors, such as Yes-associated protein (YAP)/transcriptional coactivator with PDZ-binding motif (TAZ), β-catenin, and nuclear factor kappa B (NF-κB) in cancer cells and CAFs (171). Activation of YAP1 in CAFs further promotes matrix stiffening and angiogenesis, providing a self-sustaining positive feedback loop. A recent review discusses the EV-mediated cancer-CAF intercellular crosstalk in much greater detail (84). In addition to normal fibroblasts, CAFs have also been reported to originate from several other cell types such as adipocytes, stellate cells, endothelial cells, and bone marrow-derived mesenchymal stem cells (167, 168, 172–174). CAFs are the most abundant cells in the tumor stroma and are the primary architects of the tumor microenvironment. CAF EVs not only remodel ECM but also facilitate further tumor growth by inducing microvasculature development, immune modulation, therapy resistance, and metastasis, which are discussed in subsequent sections.

#### Angiogenesis

Angiogenesis is the process of new blood vessel formation. While coordinated angiogenesis plays a critical role in developmental processes, normal growth, and repair, aberrant angiogenesis is characteristic of several pathologies including cancer. In recent years, two major concepts have emerged regarding how EVs promote angiogenesis in cancer. The first is the ability of cancer EVs to directly induce angiogenesis. Cancer EVs can either create deposits of signaling molecules in the ECM that provides temporal and spatial information for new microvasculature formation or they can be directly taken up by endothelial cells. Cancer EVs often contain various cargo that promotes angiogenesis upon endothelial cell internalization. Discussed in great detail by Zhang et al., this cargo includes proteins (VEGF_90K_, ASPH, ICAM1, CD44v5, ROCK1/2, ATF2, soluble E-cadherin) miRNAs (miR-23a, miR-130a, miR-25-3p, miR-26a, miR-182-5p, miR-21, miR-210, miR-9), lncRNAs (lncRNA-H19, lncRNA-MALAT1, lncRNA-TUG1, lncRNA-p21, lncRNA-GAS5, lncRNA-AHIF, lncRNA-HOTAIR, lncRNA-CCAT2, lncRNA-POU3F3), circular RNAs (circ-SHKBP1 and circRNA-100338), and others (99). Secondly, cancer EVs can reprogram different stromal cell types such as fibroblasts, macrophages, and mesenchymal stem cells to CAFs (discussed above), which in turn promote angiogenesis via releasing proangiogenic secreted factors such as VEGF, PDGF, and TGF-β, and by altering tumor mechanics (175–178). Recent studies have also reported release of proangiogenic cargo in CAF-derived EVs such as miR-10a-5p, miR-135b-5p, INHBA, and THBS1/2 (100–102). The establishment of new vasculature modulates immune cell infiltration and vice versa, which further influences tumor’s paracrine and endocrine crosstalk landscape (179).

A recent study demonstrated that the protein corona on the surface of EVs released by human placental stromal cells can also promote normal angiogenesis *in vivo (* 103). Although proteomic analysis identified enrichment of proangiogenic factors in placental stromal cell EVs, removing protein corona from the EVs, or replacing it with albumin, was sufficient to significantly reduce their angiogenesis potential. The EV protein corona has remained underappreciated in the field of EV and cancer biology – it remains to be seen if the extravesicular protein layer plays a critical role in mediating intercellular crosstalk, biodistribution, and in facilitating the various hallmarks of cancer.

#### Immune modulation

Immune response regulation is critical during pathogen exposure and homeostasis. For instance, lumens of epithelial tissues are under constant exposure to foreign pathogens, and unregulated lumen access can lead to severe immune response resulting in tissue damage and disease. To maintain homeostasis and immune regulation, normal epithelial cells form an intact barrier through cell-cell adhesion proteins and express ligands on their surface and the surface of their EVs (such as FasL, TRAIL, and PD-L1) that induce programmed cell death in the recipient immune cells. During barrier disruption or pathogen exposure, a signaling cascade is initiated through various immune cell types such as dendritic cells, B cells, and T cells that results in immune response activation, regulation, suppression, and memory. Immune cell types were among the first identified where biogenesis of EVs, especially exosomes, and the transfer of functional cargo was described (180–182). Since then, EVs have been shown to mediate and modulate several aspects of the immune response cascade such as antigen presentation, cytokine release, macrophage maturation, T cell activation and differentiation, and autoimmune suppression, which are thoroughly illustrated by Marar et al. (121).

Immune evasion is one of the major hallmarks of cancer. During tumor progression, EVs can regulate immune cells through two main types of interactions – (i) cancer EVs and (ii) EVs secreted by tumor-supporting cell types. Cancer EVs can modulate the activation, maturation, and differentiation of several immune cell types such as monocytes, macrophages, dendritic cells, B cells, and T cells, which are summarized by Hou et al. (183). In monocytes and macrophages, cancer EVs can activate Toll-like receptor-mediated signaling cascade, triggering NFκB- and STAT3-mediated production of proinflammatory cytokines – IL-6, IL-8, IL-1β, CCL2, G-CSF, and TNF-α (104, 105). Cancer EVs can also promote monocytes and macrophages to a pro-tumorigenic M2-macrophage state (106, 107). The acid or hypoxic microenvironment resulting from metabolic programming (discussed above) has also been linked to M2-like polarization of macrophages (108). Additionally, cancer EVs can significantly inhibit the differentiation of monocytes to dendritic cells, generating a myeloid-derived suppressive population. The suppression of dendritic cell maturation is mediated by various cargo in or on cancer EVs such as expression of human leukocyte antigen (HLA)-G, -E, and -F, heat shock proteins HSP72 and HSP105, miR-203, and dsDNA (109–112). Cancer EVs can also directly modulate T cell function. Cancer cells and their EVs often overexpress apoptosis-inducing ligands, such as PD-L1, FasL, TRAIL, NKG2DLs, and others that induce apoptosis in CD8+ T cells and NK cells and inhibit their cytotoxicity (113). PD-L1 can be present in both soluble and membrane-bound forms, and while both forms have been reported to suppress T cell function, it remains unclear which form is more active. PD-L1-mediated T cell suppression is reported to require proximity to other co-stimulatory molecules, such as TCR-CD28 and peptide/MHC complex (184). For this reason, membrane-bound PD-L1, whether on the cell surface or EV surface has been proposed to be a more potent immunosuppressant than soluble PD-L1; however, the two forms have not been compared directly for their activity (185).

EVs from tumor-supporting cell types can also promote immune evasion through mechanisms described above. The ability of dendritic cells and their EVs to mediate T-cell activation is significantly inhibited in an incomplete maturation state (116). As discussed above, cancer EVs inhibit monocyte-to-dendritic cell differentiation generating myeloid-derived suppressive population. This population plays a critical role in immune evasion by suppressing effector T cells and increasing immunosuppressive regulatory T cells (117, 186). EVs from myeloid-derived suppressive cells have also been shown to recapitulate the same functions in normal physiology and cancer (118). EVs secreted by several immune cell types, such as monocytes, tumor-associated macrophages, and dendritic cells have also been shown to express PD-L1 (119, 185). CAF EVs are perhaps the most influential in promoting immune evasion through suppression of tumor infiltration of T cells. As discussed previously, CAF EVs contribute to extensive ECM remodeling which results in dense and stiff ECM. T cell motility is dependent on chemokine gradients, and while T cells can migrate through a loose fibronectin and collagen ECM, their migration is severely reduced in dense matrix areas (187). The acidic and hypoxic tumor microenvironment further contributes to immunosuppression by reducing the activity of effector T cells (188). These tumor environmental factors severely limit tumor infiltration and activity of T cells, which contributes to immunotherapy resistance (discussed below).

Cancer EVs can also promote antitumor immunity in the presence of mature dendritic cells through expression of tumor-associated antigens, damage-associated molecular patterns, and other cargo, which is reviewed by Marar et al. (121). Furthermore, the relative fraction of soluble and EV-associated cytokines is altered upon T cell activation. In activated T cells, more cytokines are released in the free form and the EV-associated cytokines shift from an encapsulated state to a surface-attached state (125). This further highlights the importance of studying protein corona on the surface of EVs, which has been shown to mediate angiogenesis and immunomodulation (103). We currently do not have a comprehensive understanding of the immunosuppressive EV subpopulations and if these subpopulations share a common biogenesis. For instance, EVs expressing PD-L1, FasL, or others are considered to bear immunosuppressive functions, but a comprehensive understanding of their composition and biogenesis will be crucial to develop therapies that directly target these immunosuppressive subpopulations or inhibit their biogenesis.

#### Therapy resistance

Conventional therapies to treat cancer, such as surgery, chemotherapy, and radiation are often associated with therapeutic resistance. The development of resistance is attributed to tumor growth kinetics, tumor heterogeneity, cellular metabolic changes, genetic mutations, epigenetic modifications, undruggable genetic drivers, immune evasion, and others, as illustrated here (189). In fact, even targeted immunotherapies that have had great success, such as PD-1 blockade therapy, can result in acquired resistance by loss of β2-microglobulin (which impairs tumoral antigen presentation) and JAK1 or JAK2 mutations (which render tumor cells insensitive to INF-γ) in melanoma (190). While several mechanisms have been proposed to contribute to drug resistance, which are discussed in-depth by Zaretsky et al. (189), we will highlight emerging roles of EVs in the drug resistance process.

EVs have been shown to contribute to drug resistance through four broad mechanisms – (i) EV-mediated drug export/efflux; (ii) cargo transfer to sensitive cells; (iii) EV sequestration of the drug, and (iv) EVs contribution to physical barriers. A few early studies described EV-mediated active/passive export that facilitates resistance against mitoxantrone and cisplatin (126, 127). This export is mediated by transporter proteins, including ABCG2, ABCA3, and P-glycoprotein. However, functional cargo transfer from resistant cancer cells, cancer stem cells, or tumor-supporting cell types to sensitive cancer cells has been described as the major mechanism of EV-mediated drug resistance. EV-mediated transfer of proteins (MRP1, UCH-L1, PDGFRβ, PTPRZ1-MET fusion protein), miRNAs (miR-222, miR-96, miR-100-5p, miR-146a-5p, miR-155), and lncRNAs (lncUCA1, lncARSR) have been reported to confer resistance to several anti-cancer drugs such as Adriamycin, docetaxel, gemcitabine, tamoxifen, cisplatin, the BRAF inhibitor PLX4720, and temozolomide, which was summarized by Namee et al. in a recent review (128). Resistance to 5-fluorouracil in colon cancer was attributed to increased EV-mediated export of the tumor suppressive miRNAs miR-145 and miR-34a (114). Recently, EV secretion of metabolic enzymes such as ALDOA, ALDH3A1, and PKM2 by irradiated or drug-resistant lung cancer cells has been described to metabolically reprogram recipient cells to increase glycolysis. The metabolic shift results in high amounts of reductive metabolites that neutralize radiation- or cisplatin-induced reactive oxygen species, suppress apoptosis, and enhance migration and invasion in recipient cells (115, 129, 130). Functional transfer of EV cargo from tumor-supporting cells can also promote drug resistance. CAF EVs can transfer Annexin A6, miR-21, miR-92a-3p, lncRNA H19, lncRNA CCAL to promote resistance to paclitaxel, cisplatin, and oxaliplatin in various cancers (131–134, 191). Furthermore, CAF EVs can also support proliferation of cancer stem cells – a population inherently resistant to chemotherapy and other therapies (192, 193). Tumor-associated macrophages confer cisplatin resistance in gastric cancer via EV-mediated transfer of miR-21 (135). Mesenchymal stem cell EVs have also been shown to promote resistance against 5-fluorouracil by activating Raf/MEK/ERK pathway in cancer cells (194). In contrast to CAF EVs, EVs from normal mesenchymal stromal cells enhance sensitivity of myelogenous leukemia cells to Imatininb – a tyrosine kinase inhibitor targeting BCR-ABL (195). It is not yet clear whether EVs from normal stromal cells can also enhance drug sensitivity in solid tumors.

EVs can also sequester the anti-cancer drug. For instance, in an anti-CD20 humoral immunotherapy in B-cell lymphoma, EVs released by cancer cells also expressed CD20 and sequestered anti-CD20 antibodies mediating resistance to immunotherapy (137). Similarly, EVs can also sequester tyrosine kinase inhibitors by virtue of tyrosine kinase expression on EV surface, modulating sensitivity to therapies such as anti-HER2 Transtuzumab and Lapatinib (120). Extrapolating from these studies, EVs can potentially reduce the efficacy of anti-PD-L1 immunotherapies as immunosuppression in several cancers has been linked to PD-L1 expression on EVs (185). Anti-PD-1 immunotherapy has been proposed as an effective alternative in such a scenario. The contribution of EVs to increased ECM density, tumor stiffness, and acidic and hypoxic tumor microenvironment hinders effective distribution of anti-cancer drugs within tumors and plays a role in radiation resistance (170, 196). The remodeled ECM also renders immunotherapies ineffective due to the inability of T cells to perform their effector functions or infiltrate the tumor stroma altogether. Furthermore, certain drugs which have a high affinity for collagen-rich ECM, such as cisplatin, may have undesirable biodistribution as tumor ECM can often be fibronectin-rich (197).

The above body of literature signifies EVs as key mediators of major hallmarks of cancer during tumor progression and survival. Their ability to mediate crosstalk across various cell types as well as across acellular microenvironments through autocrine and paracrine signaling loops facilitates a tumor microenvironment that ensures tumor growth, survival, and resistance to therapies.

### Metastasis

Metastasis is defined by successful colonization of cancer cells in an organ or tissue distant to the primary origin site. Over the past 150 years, several hypotheses were proposed for metastatic occurrence, however, Stephen Paget’s hypothesis of ‘seed and soil’ proposed in 1889 is now widely accepted (198). To paraphrase the hypothesis, it stated that ‘while cancer cells can circulate throughout the body, they will only proliferate where the microenvironment is favorable’. After being debated for over a century, the hypothesis received seminal support in 1980 when Hart et al. provided definitive proof that although melanoma tumor cells reached the vasculature of all organs, metastasis developed in orthotopic and grafted lungs and ovaries, but not in kidneys or other organs (199). Subsequent studies investigating organ-specific metastasis focused largely on the intrinsic properties of the cancer cells, such as genes, surface receptors, and pathways regulating colonization, in directing organotropism. However, through recent studies, we are beginning to understand the features of the secondary homing sites and the distant cancer-organ crosstalk that regulates pre-metastatic niche formation in distant organs, increasing the likelihood of successful metastasis. EVs, due to their ability to form receptor-ligand interactions as well as carry soluble cargo, are considered central to the endocrine signaling process between the primary cancer and secondary organs during metastasis. Here, we will present findings from recent studies on how EVs contribute to pre-metastatic niche formation by reprogramming and remodeling the secondary organ sites.

Perhaps, the most notable recent study that demonstrated how EVs direct, and redirect, organ-specific metastasis is of Hoshino et al (23). The study showed that EVs from lung-, liver-, and brain-tropic tumor cells are preferentially taken up by resident cells at their predicted destination, i.e., lung fibroblasts and epithelial cells, liver Kupffer cells, and brain endothelial cells, respectively. Interestingly, the organotropism displayed by EVs was attributed to their integrin expression profiles. Lung-tropic EVs were enriched in ITGα_6_ and its partners ITGβ_1_ and ITGβ_4_, whereas ITGβ_5_ and ITGα_v_ were enriched in liver-tropic EVs (23). Furthermore, treatment with EVs from lung-tropic tumor cells remarkably enhanced the lung metastatic capacity of bone-tropic tumors, highlighting their ability to redirect metastasis. In addition to specific biodistribution, EVs from lung-tropic tumors reprogrammed lung fibroblasts by upregulating S100 family of genes and increasing Src phosphorylation to promote a promigratory and proinflammatory phenotype (23). As fibroblasts are the primary architects of ECM, EV-mediated molecular reprogramming of resident fibroblasts is suggested to be responsible for the drastic changes in mechanostructural properties, such as elasticity and stiffness, of lung and liver tissues following treatment with breast cancer EVs (200). These EV-mediated molecular and biomechanical changes in normal tissues further contribute to the proinflammatory microenvironment, leading to recruitment of bone marrow-derived progenitor cells. In metastatic melanoma, the progenitor cells have been shown to undergo reprogramming via tumor EV-mediated MET signaling (139). The reprogrammed bone marrow-derived cells and the inflammatory microenvironment support pre-metastatic niche formation through several processes, such as leaky vasculature induction, ECM remodeling, immunosuppression, and others. Furthermore, EVs secreted by bone marrow-derived cells contain pro-metastatic cargo, such as miR-92a, that activates hepatic stellate cells, increasing ECM deposition. The remodeled liver microenvironment enhances accumulation of immunosuppressive cells and cancer cell attachment and colonization, thereby promoting liver metastasis of lung cancer (140). In another study, EV-containing CEMIP (cell migration-inducing and hyaluronan-binding protein) has been shown to promote metastasis of breast cancer cells to the brain. CEMIP positive EVs were taken up by brain endothelial and microglial cells, which resulted in endothelial branching and upregulation of pro-inflammatory cytokines in the perivascular niche (138). Gastric cancer EVs have also been implicated in peritoneal metastasis due to their ability to upregulate fibronectin and laminin in mesothelial cells, increasing attachment between cancer cells and mesothelial cells (201, 202).

The above studies posit EVs as critical mediators of endocrine crosstalk during pre-metastatic niche establishment and metastasis. However, several aspects of the underlying biology remain unclear. For instance, the biological ‘switch’ that determines when to switch the primary tumor-supporting EV secretion to a pro-metastatic EV release is not clear. One of the mechanisms proposed to mediate the metastatic ‘switch’ is the establishment of cancer stem cells during cancer progression. Indeed, EVs from melanoma stem cells can transfer their metastatic ability to low-metastatic melanoma cancer cells (203). This would indicate that while the primary cancer cell EVs are involved in tumor growth and survival, establishment of cancer stem cells initiates release of a pro-metastatic EV subpopulation which must reach a certain threshold to instruct the underlying biological steps of metastasis. Further studies are needed to elaborate these aspects of EV biology in cancer metastasis, and the findings may present significant clinical potential as metastasis continues to be the major cause of cancer-related deaths (204, 205).

## Perspectives on EV-based cancer therapies

EVs have gained much attention as potential vehicles for the delivery of anti-cancer therapeutics for several reasons – (i) their ubiquity in biological fluids and ability to carry functional cargo, (ii) their superior biocompatibility and low immunogenicity, (iii) their ability to target cells via receptor-ligand interactions, and (iv) their enhanced retention in circulation and ability to cross biological barriers, such as the blood-brain-barrier. Several approaches have been used to load therapeutically active cargo into EVs. The most common *in vitro* method is passive mixing of the drug (for example, curcumin, acridine orange, doxorubicin, or paclitaxel), with isolated EVs, or active loading via electroporation. In a phase I clinical study (NCT03608631), mesenchymal stem cell EVs loaded with KRAS^G12D^ siRNA via electroporation are currently being evaluated as a therapeutic for the treatment of pancreatic ductal adenocarcinoma (206, 207). Direct treatment of the source cell before EV isolation has also been used to promote enrichment of the drug in EVs. Another approach relies on genetic engineering of EV-producing cells to overexpress proteins (TRAIL), a miRNA (miR-122), or a mRNA (e.g., protein–cytosine deaminase (CD) fused to uracil phosphoribosyltransferase). EV-producing cells can also be genetically engineered to produce EVs that express specific surface proteins or peptides for targeted delivery to cancer cells, which has been extensively reviewed by Abdelaal et al. in the context of RNAi therapeutics (208). Genetically engineered tumor cells have also been used to overexpress immune response-inducing antigens (for example, ESAT-6 from *Mycobacterium tuberculosis* and immunostimulatory CpG DNA) (209, 210). Alternatively, EVs from non-human sources, such as bovine milk and plants, are also being evaluated for their ability to deliver cancer therapeutics (211, 212). For example, oral administration of curcumin-loaded plant-derived EVs is currently being assessed for delivery to colon cancer patients in a phase I clinical trial (NCT01294072) (213). For a comprehensive list of EV-associated drug delivery vehicles and clinical trials in cancer, the reader is guided to a recent review by Xu et al. (214).

Despite these advances in EV-based cancer therapeutics and their future potential, there are several challenges that need to be addressed, including long-term safety, that need to be addressed. Firstly, EV heterogeneity is a significant challenge in the field of EV biology. EVs are a heterogenous population of vesicles, and their secretion and content dynamically change based on the growth conditions. For therapeutic applications, even if small EVs are enriched based on their size, they represent multiple biogenesis origins and diverse surface and luminal cargo, which can have unintended effects. Secondly, the process of genetically engineering EV-producing cells to overexpress a therapeutic cargo (protein, miRNA, or mRNA) can itself alter EV secretion, which requires comparing the safety and efficacy of genetically engineered EVs to the EVs from parent cells. Thirdly, direct loading of therapeutic cargo into EVs via transfection or electroporation results in lower loading efficiency, resulting in the necessity for higher doses of EVs which could lead to dose-associated toxicity. Furthermore, due to the inability to efficiently scale up the process, direct loading techniques pose their own challenges for high-throughput applications. Lastly, recent studies have indicated that EVs have a protein corona on their surface that can contribute to angiogenesis and immunomodulation (103). As of now, it is unclear whether the protein layer positively or negatively influences the therapeutic application of EVs. The protein corona, which could be responsible for enhance retention of EVs in circulation (207) or could contribute to unintended off-target effects, needs to be taken into consideration for future EV therapeutic approaches. Regardless, EVs represent a biocompatible mode of drug delivery that has several advantages over other delivery approaches. Further understanding of EV biology, such as their heterogeneity, protein corona, biogenesis, and cargo loading mechanisms, will significantly contribute to the development of EV-based therapeutics in cancer and other diseases.

## Conclusion

In the past 40 years, our understanding of EVs have undergone a paradigm shift from being considered as ‘waste bags’ to central mediators of cell-to-cell signaling. This is underscored by their ubiquitous nature in every biological fluid, ability to cross biological barriers, and contain cargo that indicates the pathophysiological state of an organism. While individual signaling molecules, such as growth factors, secreted proteins, RNAs, DNAs, and others, are considered important in cell-to-cell communication, EVs have emerged as ‘signaling vesicles’ that can collectively deliver diverse information to recipient cells. Recent studies have demonstrated that EVs are highly heterogenous in terms of their size, biogenesis, cargo, and function. While ‘exosomes’, ‘microvesicles’ and ‘apoptotic vesicles’ have been known, new subtypes have emerged in recent years, such as ‘mitovesicles’ that are proposed to originate from mitochondria, ‘migrasomes’ that are formed during cell migration, ‘exophers’ that are large, shed vesicles critical for cellular homeostasis, ‘megavesicles’ that are atypically large vesicles enriched in metabolic enzymes and EVs released during necrosis, necroptosis, ferroptosis, and pyroptosis. In addition, recent studies have also identified extracellular nanoparticles that are devoid of a lipid bilayer, called ‘exomeres’ and ‘supermeres’. These newly identified nanoparticles have a relatively higher abundance of miRNAs and miRNA-binding proteins, such as AGO1-4. It is important to note that none of the existing EV isolation methods can segregate heterogenous EVs into individual pure subpopulations, which creates a significant challenge in understanding their biogenesis and functional effects. Furthermore, several EV subpopulations share similarities in their biogenesis, such as ESCRT complex utilization in exosome and microvesicle biogenesis, mitochondria presence and double membrane structures of mitovesicles and migrasomes, and similarities between exophers and EVs derived from secreted autophagy. EVs released during other emerging forms of programmed cell death such as ferroptosis, pyroptosis, and necroptosis, and their underlying molecular mechanisms are significantly understudied.

EVs can mediate various hallmarks of cancer that support tumor growth, survival, and metastasis. However, much less is known about whether and how EVs contribute to cancer initiation, early mutational landscape, and precancerous inflammation. For instance, do EVs secreted by early microtumors provide a competitive advantage through autocrine signaling or by influencing the growth of adjacent normal epithelium is a question that largely remains unanswered. Similarly, it is not known if EVs play an important role in immune suppression or evasion during initiation stages of hematologic malignancies. Precancerous inflammation, especially in colitis-induced colorectal cancer, is mediated by intercellular crosstalk between gut microbiota, colon epithelial cells, and macrophages (78). However, the role of EVs in precancerous inflammation crosstalk has not been fully elucidated. Conversely, the role of EVs in tumor growth and survival has been extensively studied regarding metabolic reprogramming of tumor-supporting cell types, ECM remodeling, angiogenesis, immune modulation, and therapy resistance. In some instances, EV-mediated biology is proposed to be more efficient over conventional signaling, such as (i) membrane-bound metalloproteinase secretion in nano-sized EVs allows better diffusion through the ECM, (ii) the ability of EVs to bind to ECM proteins via surface receptors provides spatial information for new vasculature formation, and (iii) the ability of EV membrane-bound PD-L1 to mediate immunosuppression due to presence of co-stimulatory proteins on the same membrane. As EVs are a mixture of heterogenous subpopulations, it remains unclear if specific EV subpopulations are responsible for effects on ECM, microvasculature formation, or immune modulation and if these subpopulations have unique intracellular biogenesis or trafficking. Understanding this heterogeneity and their underlying biogenesis will allow us to inhibit or target specific EV subpopulations and the effects mediated by them.

EVs are ideal endocrine signaling mediators due to their ubiquitous nature in biological fluids, low immunogenicity, enhanced retention in circulation, and ability to carry and protect diverse functional cargo, cross biological barriers, and interact with target cells via receptor-ligand interactions. These properties are also central to pre-metastatic niche establishment and metastasis. Metastatic cancers have increased EV secretion, altered EV cargo, and the EV biodistribution phenocopies the eventual metastatic spread. EVs can reprogram fibroblasts and bone marrow progenitor cells at the secondary sites, altering the viscoelastic properties of the tissue and promoting immunosuppressive microenvironment. The altered ECM properties allow cancer cells shed from the primary tumor to efficiently ‘stick’ to the tissue and the immunosuppressive environment facilitates colonization. While the ‘switch’ from primary to metastatic state requires further investigation, emergence of cancer stem cells is proposed to be an inflection point during cancer progression. While EVs from cancer stem cells can enhance the metastatic capability of low metastatic cancers, further studies are required to elucidate the metastatic ‘switch’ hypothesis and the underlying EV biology. The biological features of EVs mentioned at the beginning of the paragraph also make them ideal for cancer therapeutics advancement. However, the heterogeneous population, diverse surface and luminal cargo, understudied surface protein corona, long-term safety and technical challenges associated with large scale EV production are some of the challenges that would need to be resolved to bring EV-based therapeutics to the clinic.

## Author contributions

IS drafted the article with assistance from AK. IS and AK edited the manuscript. IS created the figures. All authors contributed to the article and approved the submitted version.
